# Willingness to receive mpox vaccine among men who have sex with men: a systematic review and meta-analysis

**DOI:** 10.1186/s12889-024-19260-9

**Published:** 2024-07-15

**Authors:** Jiajia Liu, Siying Liu, Simin Yu, Xiaoyu Du, Jiaqi Hao, Ruixue Hui, Amos Buh, Wenjun Chen, Jia Chen

**Affiliations:** 1 Xiangya School of Nursing, Central South University, 172 Tongzipo Road, Yuelu District, Changsha, Hunan China; 2 Hunan Provincial Maternal and Child Health Care Hospital, Changsha, China; 3 Centre for Social Research in Health, University of New South Wales, Sydney, Australia; 4 Ottawa Hospital Research Institute, Ottawa, Canada

**Keywords:** Mpox, Mpox virus, Vaccine, Men who have sex with men, Systematic review, Meta-analysis

## Abstract

**Background:**

Since May 2022, mpox outbreaks have been occurring in non-mpox endemic areas, with the main population affected being men who have sex with men (MSM). Outbreak prevention and control depend not only on the effectiveness of vaccines but also on people’s willingness to receive these vaccines. Currently, there is lack of synthesis on the overall rates and influence factors of MSMs’ willingness to vaccinate against mpox. Therefore, we systematically reviewed studies that assessed the willingness of MSM to receive mpox vaccine.

**Methods:**

Studies reporting mpox vaccination intentions among MSM were included by searching five databases (PubMed, Web of Science, EMBASE, CINAHL, and SCOPUS) from inception to May 12, 2024. The quality of the included literature was assessed using Joanna Briggs Institute’s critical appraisal tool. The data analysis software is Stata17. The systematic review has been registered with Prospero (registration ID: CRD42023452357).

**Results:**

Twenty cross-sectional studies were included in the review. Meta-analysis results showed that the pooled willingness rate of vaccinate against mpox was 77.0% (95% CI: 73-81%, I^2^ = 99.4%). According to subgroup analysis, study countries (*P* = 0.002), research sample size (*P* = 0.001), and whether participants were infected with HIV (*P* = 0.002) may be sources of heterogeneity. The results of the meta-analysis of influencing factors showed that more number of sexual partners (OR: 2.24, 95%CI: 1.86–2.69), pre-exposure prophylaxis use (OR: 6.04, 95%CI: 4.80–7.61), history of sexually transmitted infections (OR: 2.96, 95%CI: 2.33–3.76), confidence in the vaccine’s effectiveness (OR: 2.79, 95%CI: 2.04–3.80) and safety (OR: 10.89, 95%CI: 5.22–22.72), fear of mpox infection (OR: 2.47, 95%CI: 2.11–2.89) and epidemics (OR: 2.87, 95%CI: 2.22–3.70), high mpox knowledge (OR: 2.35, 95%CI: 1.51–3.66), and the belief that people at high risk should be prioritized for vaccination (OR: 3.09, 95%CI: 1.40–6.84) were the facilitators of vaccine willingness. In addition, as a secondary outcome, meta-analysis results showed a pooled unwillingness rate of 16% (95% CI: 13-20%, I^2^ = 98.1%, 9 studies).

**Conclusion:**

Willingness to vaccinate mpox was high among MSM, but some participants still had negative attitudes towards vaccination. Therefore, the Ministry of Public Health should develop targeted and effective strategies against those influencing factors to prevent and manage mpox outbreaks.

**Supplementary Information:**

The online version contains supplementary material available at 10.1186/s12889-024-19260-9.

## Introduction

Epidemics of infectious diseases have long been a major public health challenge globally. In May 2022, an outbreak of monkeypox (Mpox) suddenly appeared and rapidly spread to Europe, the Americas, and then to all six World Health Organization (WHO) regions [1]. Mpox is a disease caused by the mpox virus (a zoonotic virus) [2]. Human-to-human transmission of mpox can occur through direct contact with infectious skin, mouth, or other lesions on the genitals [1]. This global outbreak primarily (but not only) affects gay and bisexual individuals, but also affect other men who have sex with men (MSM) - thus establishing a steady chain of transmission from person to person among this group people [3].

Vaccination is an effective measure to reduce the spread of the mpox virus and protect the health of the community [4–6]. Given this, there has been a renewed interest globally in vaccination as a preventive measure for mpox. Currently, two mpox vaccines, ACAM 2000^®^ (smallpox or cowpox live vaccine) and JYNNEOS^™^ (cowpox virus modified strain - Ankara-Bavarian Nordic non-replicating antigen), are available as pre-exposure prophylaxis against mpox [7, 8]. Reports from several case studies have confirmed the effectiveness of these vaccines in preventing mpox [9, 10]. The WHO [1] and the Centers for Disease Control (CDC) [11] in the United States recommend that people at risk (researchers with occupational exposures, gay bisexual and other MSM, people with multiple sex partners, and sex workers) should be actively vaccinated against mpox during epidemics.

However, as with COVID-19, the effectiveness of mpox vaccines depends not only on the scientific success of the vaccine development [12], but equally on the willingness of people at risk to receive these vaccines [13]. Vaccine uptake willingness is therefore an important aspect that should be considered for mpox prevention and control [14, 15]. Nonetheless, vaccine hesitancy (refusal or delay of vaccination despite available services) remains a major problem facing vaccination uptake worldwide. In 2019, the WHO listed vaccine hesitancy as one of the top ten health threats facing the world [16]. There is now cumulative evidence on the willingness of different populations to receive the mpox vaccine, and the results show wide variation [17, 18]. MSM are one of the main affected groups by the mpox epidemic and the target audience for mpox vaccination [1]. Exploring the willingness of MSM to receive mpox vaccination and the factors that influence it can contribute to the successful design and implementation of public health strategies to control the spread of the virus.

Currently, a few studies have assessed mpox vaccination willingness in different populations [19–21]. However, to the best of our knowledge, there are no systematic reviews that have explored the overall rates of the willingness and the factors that influence the willingness to vaccinate against mpox among MSM. The purpose of this systematic review, therefore, was to synthesize results from different studies of mpox vaccination intentions among MSM, and to assess the factors influencing mpox vaccination intentions. This work provides a data-driven approach to support public health departments in providing scientifically effective mpox prevention guidance to MSM, while contributing to effective responses to potentially preventable future outbreaks of mpox or other similarly serious epidemic outbreaks.

## Methods

### Study design

This was a systematic review that adhered to the 2020 Preferred Reporting Items for Systematic Reviews and Meta-Analyses (PRISMA) guidelines. The protocol for this systematic review is registered with PROSPERO (CRD42023452357).

### Eligibility criteria

To be eligible for this review, a study had to be a descriptive or observational study, published in English, conducted on gay or bisexual individuals as well as other MSM, and report on participants willingness to be immunized with the mpox vaccine and factors influencing their willingness to take the vaccine.

Nonoriginal articles such as literature reviews, commentaries, conference abstracts, letters to the editor, case reports were excluded; Qualitative studies that did not assess participants willingness to take the mpox vaccine were also excluded from the review.

### Search strategy

We conducted searches in five electronic databases (PubMed, EMBASE, Web of Science, Scopus, and CINAHL). Our search terms included mpox, mpox virus, sexual minorities, gay men and MSM, vaccination, vaccine, willingness, attitude, intention, and hesitation. We applied Boolean logic operators and truncation characters to combine subject terms and keywords in the search formula. After devising the search strategy, two reviewers (JL and RH) conducted a thorough search of each database for publicly available literature related to willingness to receive vaccines against mpox, covering the period from the database’s inception up to May 12, 2024. The search was not restricted by publication status. The detailed search algorithms used for each database are presented in Appendix A.

### Study selection

We imported the search results into COVIDENCE ^TM^ [22] for literature screening. After automatically removing duplicates, literature screening was performed in two steps based on the eligibility criteria. First, three reviewers (JL, SL and SY) screened titles and abstracts of literature and eliminated those did not meet the inclusion criteria. Second, reviewers assessed eligibility separately by reading the full text of all potentially eligible studies. Any conflicts regarding the inclusion or exclusion of a particular article were resolved by consensus between the two senior researchers (JC, WC).

### Quality assessment

For quality assessment and risk of bias evaluation, we used the Joanna Briggs Institute (JBI) Critical Appraisal Checklist for analytical cross-sectional studies to assess included cross-sectional studies [23]. The checklist for cross-sectional studies of JBI analysis has eight items, including research inclusion criteria, definition of research subjects, research environment, validity of exposure measurements, objective evaluation criteria, confounder assessment and treatment strategies, reliability and validity of outcome measurements, and rational use of statistical analysis methods. “Yes”, “No”, “Unclear”, or “N/A” was applied to assess quality of each item in the checklist. Two researchers (SL, SY) assessed each study separately and determined the quality of the studies by summarizing the score of each item given by two researchers. Any disagreements were resolved by group consensus with the two senior researchers (WC, JC).

### Data extraction

Data extraction was performed by two authors (XD and JH). The extracted data included study characteristics and outcome measures. Study characteristics included study author, year of publication, country, date of survey collection, participants group, sample size. The outcome measures included main survey questions and vaccine willingness response options, and main results. The extracted data was checked for completeness by another author (WC). Any disagreements were resolved by consensus.

### Statistical analysis

We used Stata 17.0 software to analyze the data and calculate the pooled rates of vaccine willingness across all studies. Cochran’s Q test and I^2^ test were used to assess heterogeneity between studies. Statistical heterogeneity among studies was indicated by a *p* value < 0.1 and I^2^ > 50%. To explore the source of heterogeneity, we carried out subgroup analyses. Next, we combined the effect sizes while excluding significant clinical heterogeneity using a random effects model. The sensitivity analysis of the willingness rates of the included studies was performed by the one-by-one exclusion method to assess the stability and reliability of the results. We used Egger’s test at a *P* value < 0.01 to check for the presence of publication bias.

## Results

### Search resultsp

**Fig. 1:**
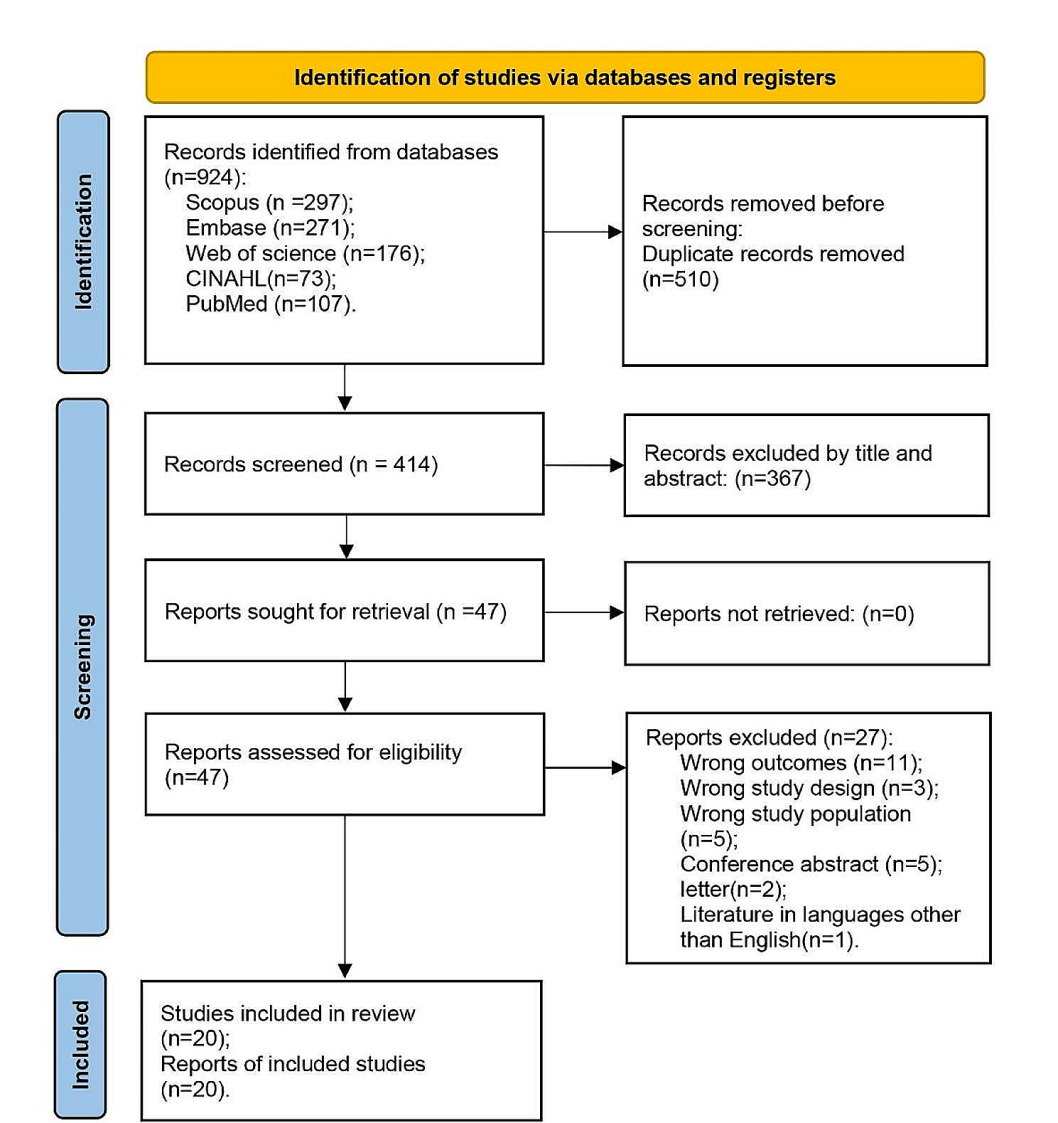
Flow diagram of the study selection process

The study search and screening flowchart is displayed in Fig. 1. A total of 924 studies were imported into COVIDENCE for screening, 510 duplicates were removed. After title and abstract screening, 47 studies were eligible for full text screening. Finally, a total of 20 studies were included in the systematic review and meta-analysis. The main reasons for full-text screening articles being excluded were studies did not evaluated willingness of vaccination (*n* = 11, 41%), they were not empirical studies (*n* = 7, 26%) or did not targeted on MSM (*n* = 5, 19%).

### Study characteristics

Researchers of all included studies conducted cross-sectional online survey in either 2022 or 2023. The Chinese population was most frequently studied, with six studies recruiting participants in China [18, 24–28], followed by three surveys in Netherlands [29–31]. Reyes-Uruena et al. conducted a survey in 59 countries or subregions in Europe [32]. Zheng et al. conducted two survey studies with different samples at two different time points [18, 24].

The sample size varied from 154 participants to 32,902 participants for each study. Four studies [26, 33–35] targeted MSM that living with HIV (MSMWH), with one study [27]included male sex workers (MSW), a subgroup of MSM. Of all studies, 11 used Likert scales as the outcome score classification standard, and seven studies rated with “yes/no”. In two studies [18, 34], participants were asked one question on vaccine hesitancy, instead of willingness. According to the JBI quality assessment tool, 11 studies were found to have a low risk of bias and 9 studies have a moderate risk of bias (Appendix B). An overview of the included studies and study characteristics is presented in Table 1.

**Table 1:** Characteristics of the included studies

Study	Country	Survey date	Participant groups	Main survey question	Survey question responses categorized as vaccination-willing	Sample size (MSM)	Willingness rate	Unwillingness rate	Risk of bias
Reyes-Uruena et al. (2022) [32]	WHO European Region^*^^1^	30 Jul-12 Aug 2022	MSM	If the vaccine for monkeypox is offered to you, will you get vaccinate?	I will get vaccinated. / Probably yes.	32,902	82.0% (26,980/32,902)	8.16% (2,686/32,902)	Low
Wang et al. (2022) [30]	Netherlands	1–15 Jul 2022	MSM	Vaccination willingness was measured using a 1–5 Likert scale (1 = very low,5 = very high). ^*2^	High. / Very high.	394	70.01% (276/394)	NR^*3^	Medium
Zheng et al. (2022) [24]	China	1–3 Jul 2022	MSM	whether they were willing to receive vaccines against monkeypox if available.	Yes.	2,618	90.20% (2,362/2,618)	9.78% (256/2,618)	Low
Zucman et al. (2022) [34]	France	Jul-Aug 2022	MSMWH^*4^	Do You Want to Get Vaccinated against MPXV?	Yes.	155	66.45% (103/155)	NR	Medium
Fu et al. (2023) [26]	China	10 Aug − 9 Sep 2022	MSMWH	Would you like to be vaccinated against mpox?	Agree. / Strongly agree.	577	56.85% (328/577)	43.15% (249/577)	Low
Li et al. (2023) [25]	China	10 Aug-9 Sep 2022	MSM	Are you willing to be vaccinated?	Quite willing to be vaccinated. / Willing to be vaccinated.	1,090	86.15% (939/1,090)	NR	Medium
Dukers-Muijrers et al. (2023) [31]	Netherlands	22 Jul-5 Sep 2022	MSM	If you could receive a vaccine against mpox, would you get vaccinated against mpox?	Yes, probably. / Yes, certainly.	1,831	81.6% (1,494/1,831)	11.63% (213/1,831)	Low
Hori et al. (2023) [17]	Japan	Sep-Oct 2022	MSM	Are you willing to receive the vaccine in the future if there is a chance to do so?	I want to be vaccinated.	732	29.2% (214/732)	NR	Medium
Chow et al. (2023) [36]	Australia	Aug-Oct 2022	MSM	Whether you intent to be vaccinated against monkeypox?	Intended to get vaccinated.	312	68.27% (213/312)	NR	Medium
MacGibbon et al. (2023) [37]	Australia	24 Aut-12 Sep 2022	MSM	If a safe and effective vaccine against monkeypox was available to you, how likely are you get vaccinated?	Likely. / Very likely.	1,733	84.07% (1,457/1,733)	NR	Medium
Svartstein et al. (2023) [35]	Denmark	1 May-31 Oct 2022	MSMWH	How willing are you to get the mpox vaccine?	High willingness. / Very high willingness.	401	57% (228/401)	NR	Low
Araoz-Salinas et al. (2023) [38]	Peru	1 Nov 2022–17 Jan 2023	MSM	Do you plan to get vaccinated against mpox when the vaccine becomes available?	I will get vaccinated. / It is likely that I will get vaccinated.	281	89.32% (251/281)	10.68% (30/281)	Low
Chen et al. (2023) [27]	China	1–31 Aug 2022	MSM	Do you plan to vaccinate against Monkeypox when the vaccine is available?	I will get vaccinated. / It is likely that I will get vaccinated.	154	63% (97/154)	37.01% (57/154)	Low
Karapinar et al. (2023) [39]	Turkey	30 Jun-12 Aug 2022	MSM	Willing to get mpox vaccine?	Yes. / No.	731	70.31% (514/731)	10.53% (77/731)	Low
Zheng et al. (2023) [18]	China	31 Jul-4 Aug 2023	MSM	If mpox vaccine is available in China, are you willing to get vaccinated?	Unwilling to take the non-free vaccine or the free vaccine.	7,538	94.41% (7,117/7,538)	NR	Low
Smith et al. (2023) [40]	UK	5 Sep-6 Oct 2022	MSM	We asked participants how likely they would be to have a smallpox vaccine if they were offered one.	Vaccinated. / Probably would be vaccinated. / Definitely would be vaccinated.	2114	SG:85% (210/247) Ginder:93.5% (777/831) Meta:96.3% (998/1,036)	NR	Low
Jongen et al. (2023) [29]	Netherlands	9 Jul-11 Aug 2022	MSM	If you could get vaccinated against mpox, would you?	Questions were assessed on a 7-point Likert scale ranging from 1 (low) to 7 (high). 1 = Definitely not, 7 = Absolutely will. ^*5^	492	77% (380/492)	NR	Medium
Andersen et al. (2024) [33]	USA	Aug 2022 - Jan 2023	MSMWH	Have you received the monkeypox vaccine?	Yes. / No but I plan to.	166	76.51% (127/166)	23.5% (39/166)	Medium
Huang et al. (2024) [28]	China	Aug-Sep 2022	MSM	Willingness to receive monkeypox or smallpox vaccination when most of your friends or sexual partners have been vaccinated.	Willing.	1,093	95.5% (1,044/1,093)	4.5% (49/1,093)	Low
Ogaz et al. (2024) [41]	UK	Nov-Dec 2022	MSM	If offered, to what extent would you be willing to be vaccinated against monkeypox?	Would be likely to get vaccinated. / Would definitely get vaccinated.	559	75% (421/559)	NR	Medium

*1: WHO European Region: The research subjects’ residence Countries or European subregions include Norway, Monaco, Portugal, Denmark, Sweden, Netherlands, Ireland, Latvia, Spain, Belgium, Austria, Luxembourg, Iceland, France, Germany, Israel, Italy, Andorra, Finland, Malta, Estonia, Baltics, Liechtenstein, Lithuania, Croatia, Greece, Poland, Romania, Turkey, Russia, Kyrgyzstan, Montenegro, Slovenia, Uzbekistan, Azerbaijan, Georgia, Armenia, Kazakhstan, Slovakia, Hungary, Ukraine, Moldova, Serbia, Albania, Tajikistan, Cyprus, Bulgaria, Belarus, Czechia, United Kingdom, Northern Europe, Western Europe, Switzerland, North Macedonia, Central Europe, Eastern Europe, Mediterranean Europe, South-East Europe, Bosnia and Herzegovina

*2: The study did not report outcome survey questions

*3: NR: No reported results

*4: MSMWH: Men who have sex with men and are living with HIV

*5: The study not described which options are identified as willing to vaccinate

### Mpox vaccine willingness rate

**Fig. 2:**
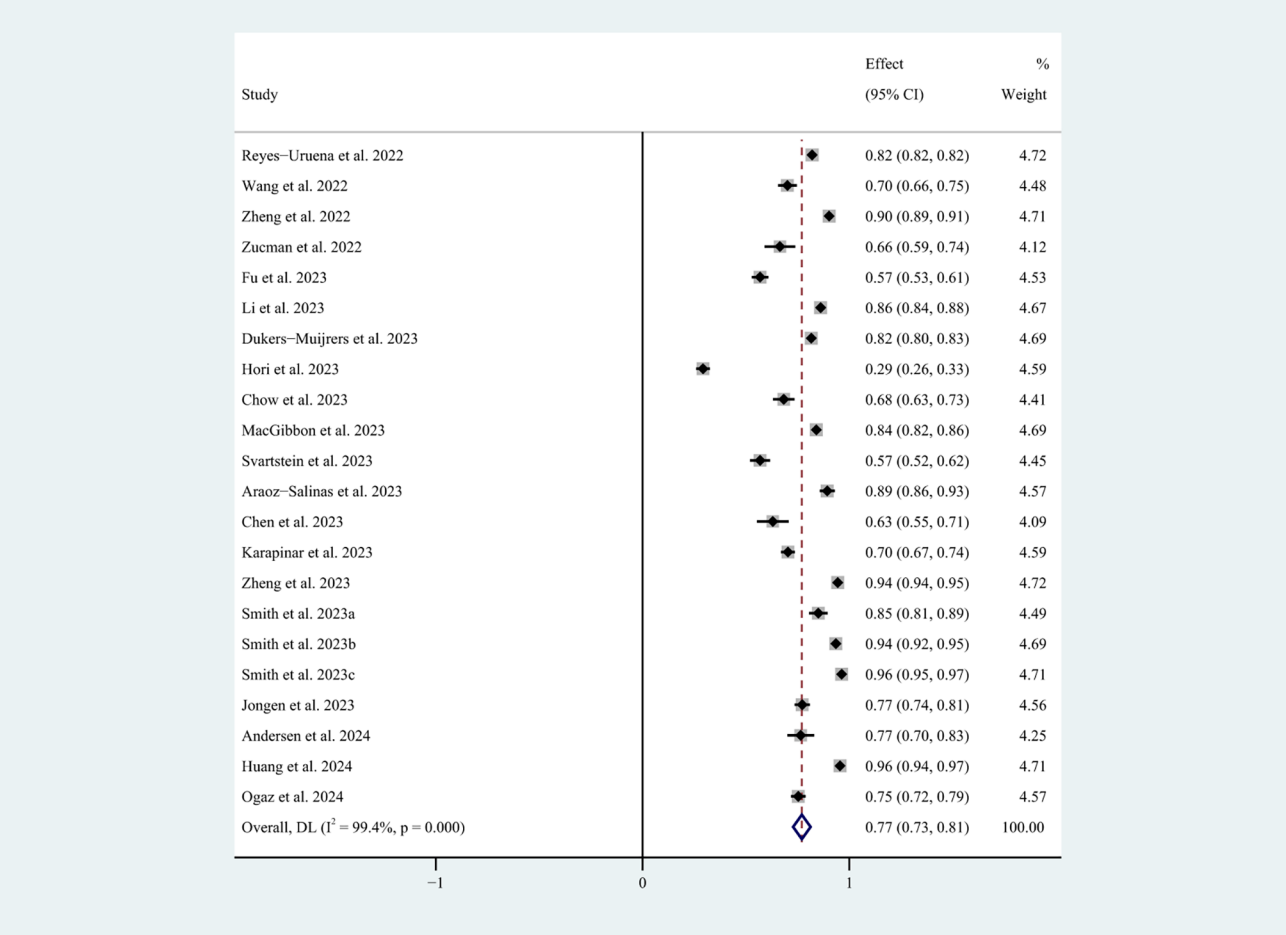
Mpox vaccine willingness rates among MSM

**Fig. 3:**
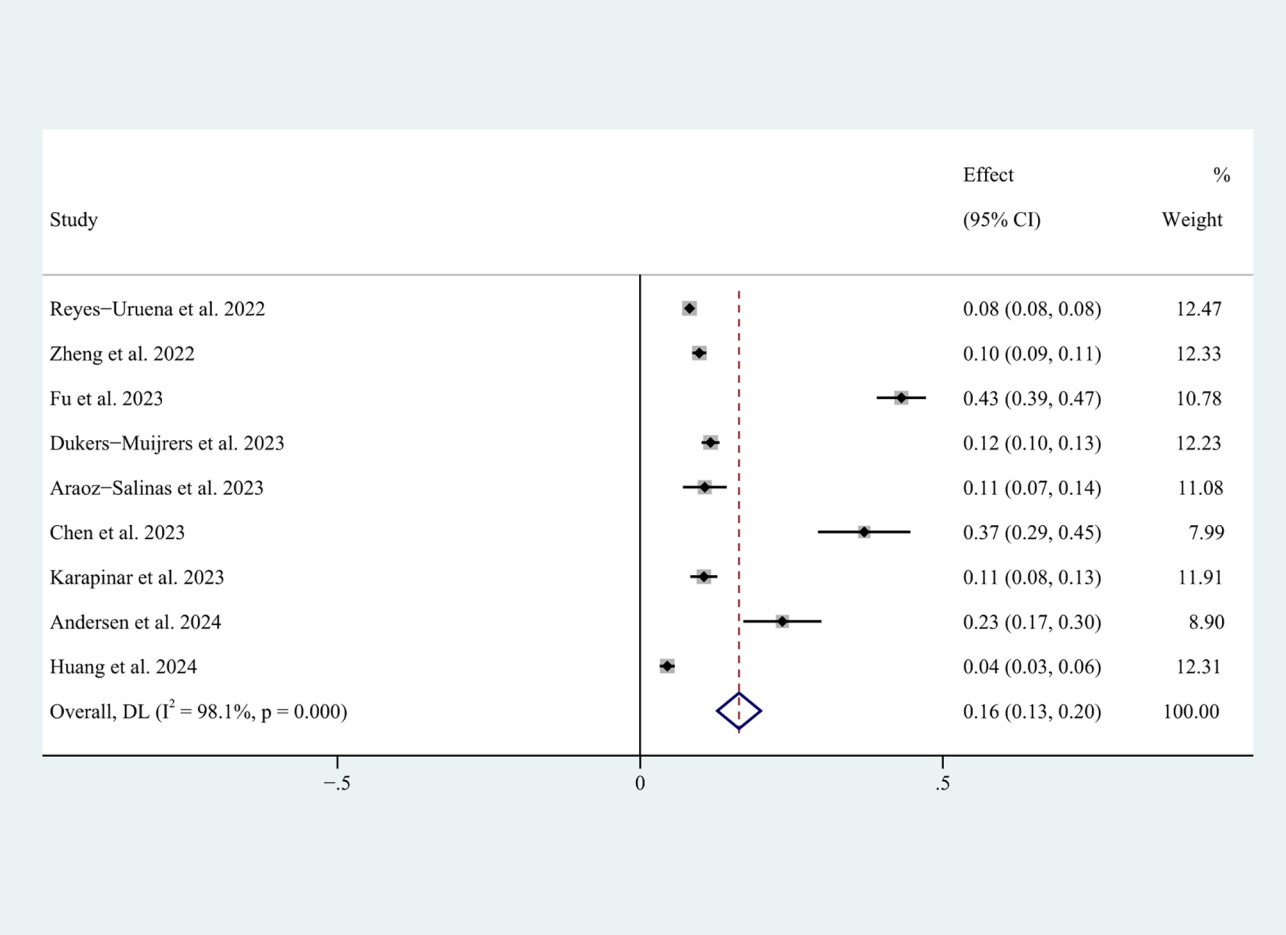
Mpox vaccine unwillingness rates among MSM

The lowest willingness rate to vaccinate reported was 29.2% (214/732) by Hori et al. [17]. The highest rate was 94.5% (1,033/1,093) as reported in Huang et al.’s study [28]. The pooled willingness rate among the 20 studies was 77.0% (95% CI: 73-81%) (Fig. 2). The I^2^ of the 20 studies was 99.4%, *p* < 0.001, which showed high heterogeneity. In addition, the pooled rate of mpox vaccine unwillingness among MSM was 16% (95% CI: 13-20%, I^2^ = 98.1%) among the included nine studies [24, 26–28, 31–33, 38, 39] (Fig. 3).

**Fig. 4:**
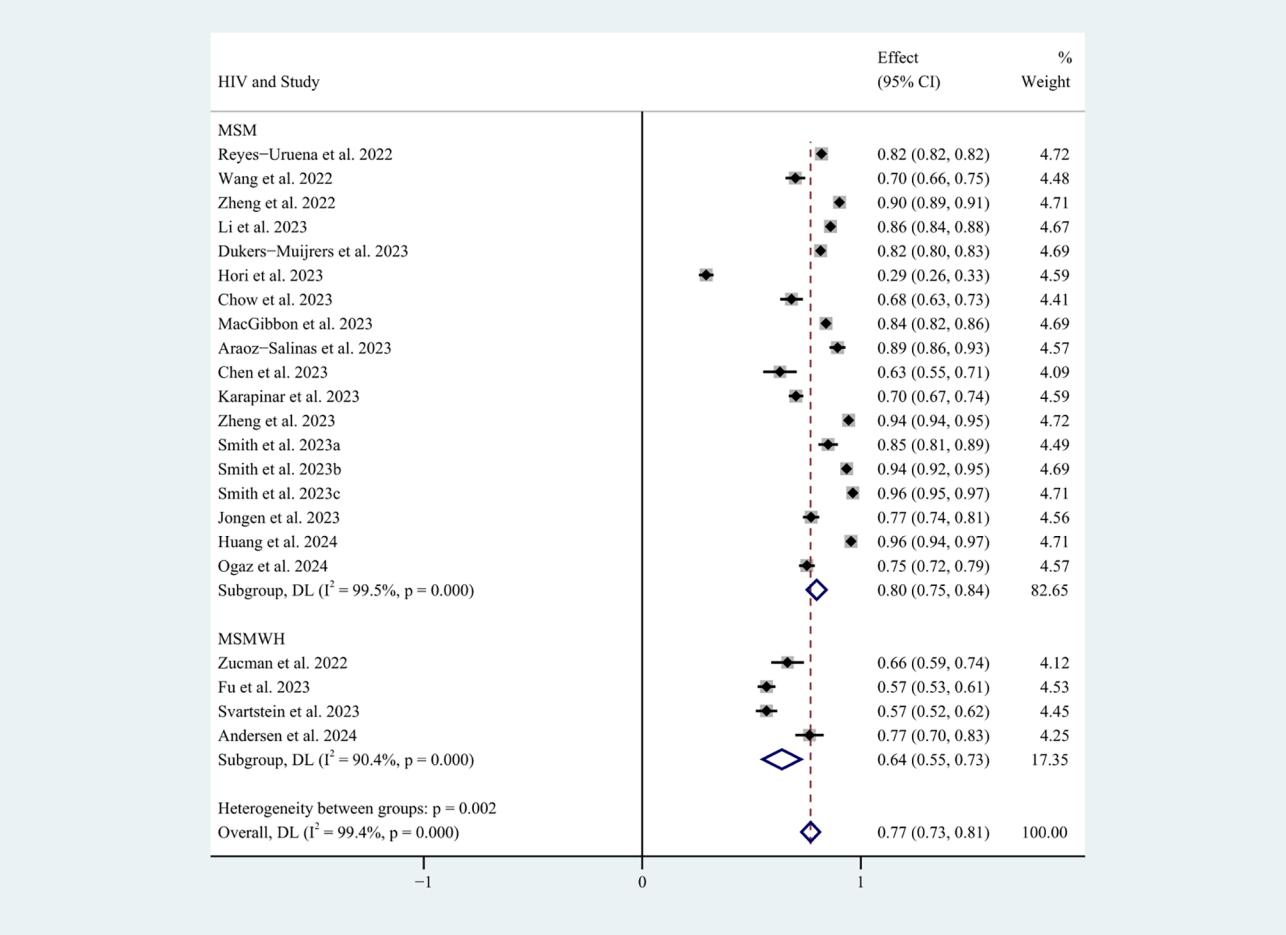
Subgroup analysis of mpox vaccination willingness rates by participant groups

Subgroup analyses were performed using survey countries (*P* = 0.002) and continents (*P* = 0.05), survey date (*P* = 0.296), study sample size (*P* = 0.001), and whether participants were infected with HIV (*P* = 0.002) as different group variables (Appendix C). The results suggest that countries, study sample size, and whether participants were infected with HIV may be sources of heterogeneity. Subgroup analysis by participant groups showed that the general MSM (80%, 95%CI: 75-84%) had a higher overall willingness rate than MSMWH (64%, 95%CI: 55-73%) (Fig. 4).

The sensitivity analysis showed that the result was stable. The result of the Egger’s test was *p* = 0.106, indicated no publication bias (Appendix C).

### Factors associated with mpox vaccine willingness among MSM

A total of 11 articles were included in the meta-analysis of factors associated with mpox vaccination willingness among MSM. Table 2 shows the factors associated with willingness, factor effect values on willingness to vaccinate, I^2^ values and H values. A total of nine factors were extracted from the meta-analysis, of which six factors (more sexual partners [26, 30, 34, 41], history of sexually transmitted diseases [35, 36, 41], use of PrEP [36, 41], worried about mpox epidemic [26, 29], Worried about mpox infection [24, 29, 30, 34], confidence in vaccine safety [26, 28]) were highly heterogeneous (I^2^ ≥ 50%), and therefore a random effects model was chosen. A fixed-effects model was applied to three factors (confidence in vaccine effectiveness [26, 29, 40], high level of mpox knowledge [24, 36], agreed that people at high-risk should be given priority for vaccination [27, 28]) that had low heterogeneity (I^2^ < 50%). All the 9 factors had statistical significance with MSM vaccination willingness, and all were facilitators of vaccination willingness.

**Table 2:** Meta-analysis of factors of mpox vaccine willingness among MSM

Factor(s)	Number of studies	I^2^ [95%CI]	Effect model	Pooled effects		H [95% CI]
				OR [95% CI]	*P*-value	
More sexual partners	4	91.8% [83.9%, 94.9%]	Random	2.24 [1.86, 2.69]	*< 0.001*	3.49 [2.49, 4.42]
PrEP use^*1^	2	87.2% [0.0%, 95.2%]	Random	6.04 [4.80, 7.61]	*< 0.001*	2.80 [1.00, 4.58]
STD^*2^	3	69.5% [0.0%, 89.0%]	Random	2.96 [2.33, 3.76]	*< 0.001*	1.81 [1.00, 3.01]
Confidence in vaccine effectiveness	3	28.1% [0.0%, 79.7%]	Fixed	2.79 [2.04, 3.80]	*< 0.001*	1.18 [1.00,2.22]
Confidence in vaccine safety	2	57.8% [0.0%, 89.8%]	Random	10.89 [5.22, 22.72]	*< 0.001*	1.54 [1.00, 3.13]
Worried about mpox infection	4	94.3% [89.2%, 96.4%]	Random	2.47 [2.11, 2.89]	*< 0.001*	4.20 [3.04, 5.28]
Worried about mpox epidemic	2	94.6% [79.8%, 97.3%]	Random	2.87 [2.22, 3.70]	*< 0.001*	4.30 [2.22, 6.14]
High level of mpox knowledge	2	35.6% [0.0%, 86.4%]	Fixed	2.35 [1.51, 3.66]	*< 0.001*	1.25 [1.00, 2.71]
Agreed that people at high risk should be given priority for vaccination	2	0.0% [0.0%, 80.1%]	Fixed	3.09 [1.40, 6.84]	0.01	0.74 [1.00, 2.24]

*1:PrEP use: HIV: Use of Tenofovir fumarate (TDF)/ Emtricitabine (FTC) and FTC/ Tenofovir elphenolamine as pre-exposure prophylaxis (PrEP) to reduce the likelihood of HIV infection in people with more risk of MSM, TG, etc

*2:STD: Participants who had been infected with a sexually transmitted disease(STD) in the last two years. STDs include, but are not limited to, syphilis, gonorrhea, genital chlamydia trachomatis infection, genital warts, genital herpes and AIDS

## Discussion

This systematic review and meta-analysis of 20 studies estimated that the willingness to vaccinate against mpox among MSM was 77.0% (95% CI: 73-81%). We found that willingness in MSM was higher than that of healthcare workers (willingness rate 58.5%, 95% CI: 40.5–67.4%) [21]. Both MSM and health care workers are at risk for mpox exposure. The majority of reported cases of mpox outbreaks are from MSM and those who are sexually active or have frequent intimate contacts [42, 43]. MSM are more likely to have a need for and priority to receive vaccination.

There are differences in willingness rates across countries. The large survey conducted by Reyes-Urueña et al. [32] revealed that people living in the subregions of South-Eastern and Central Europe as well as Eastern Europe reported lower vaccination rates than those in other regions. In addition, we found that willingness rates ranged from 56.85 to 94.5% in the six studies conducted in China [18, 24–28]. Geographic differences in vaccine willingness outcomes are plausible due to differences in government measures, social environments, or economic levels across countries. Besides, different vaccine willingness measurement tools also had an impact on the results [44]. Standardized vaccine willingness assessment tools can help improve the ability to measure, evaluate, and compare across jurisdictions and over time [45]. In 2015, Larson and the WHO Strategic Advisory Group of Experts (SAGE) on Immunization developed a tool to measure vaccine hesitancy [46]. In addition, there are other vaccine willingness assessment tools [44, 47, 48]. However, these tools have been evaluated and validated in only a few studies, or not at all. This may be the reason why included studies do not use a uniform assessment tool.

Contrary to our hypothesis, subgroup analysis results showed that compared to general MSM, the vaccine acceptance rate was lower among MSMWH (MSM with HIV) [26, 33–35]. We proposed two possible reasons: (1) Bias caused by clinical heterogeneity factors such as sample size, number of studies included, and study design. (2) Concerns about vaccine side effects may be inhibiting MSMWH’s willingness to vaccinate. For example, ACAM2000^®^ may cause severe mpox-related diseases in immunocompromised people and is contraindicated in people with HIV [49]. JYNNEOS™ has been approved by the Food and Drug Administration (FDA) and has been assessed as safe and effective for people living with HIV (PLHIV) [50, 51]. This helps to increase the tendency of MSMWH to be vaccinated. Contrary to our results, multiple studies have shown higher vaccine acceptance rates among people living with HIV compared to those without HIV [31, 37, 38, 52]. This could be explained by HIV infection making MSM more concerned about their health, leading to higher willingness to receive the mpox vaccine.

In meta-analysis of influencing factors, the number of sexual partners has a significant influence on vaccine willingness. MSM who have more sexual partners or are in open relationships show a higher willingness to receive vaccination. This contrasts with the findings of Zhao et al. [53], who explored the acceptability of human papillomavirus (HPV) vaccination among MSM. Having more sexual partners is one of the high-risk sex behaviors. As the number of sexual partners and close contacts increases, the likelihood of contracting mpox from an external source increases exponentially [54]. Therefore, public health centers can collaborate with community organizations during vaccination to provide effective sex education to MSM, thereby increase their self-protection awareness.

There is a statistically significant relationship between participants having a history of sexually transmitted diseases (STDs) and vaccine willingness. Meta-analysis results indicate that being diagnosed with at least one STD other than HIV in the past two years is associated with a higher willingness to receive vaccination [17, 24, 37, 38]. We argued that contracting sexually transmitted diseases prompts MSM to seek sexual health knowledge and guidance from healthcare professionals, leading to more attention to bodily health and willingness to receive vaccinations [38].

High level of mpox knowledge and perceived risk of mpox increase vaccination willingness among MSM. Participants who feared a mpox epidemic and avoided being infected with mpox were more likely to support vaccination. Mpox vaccine acceptance was higher among participants with high levels of mpox knowledge and those who agreed that people at high risk had priority for vaccination. These results can be explained by the health belief model (HBM). The HBM suggests that peoples’ self-perceptions of disease susceptibility and severity, as well as their perceived benefits and barriers to implementing behavior change, self-efficacy, and cues for action, strongly influence their adoption of health behaviors [55]. Public health departments can disseminate authoritative information on mpox prevention guidelines, self-care and vaccination through multiple channels in vaccination promotion programs [56].

Trust in the safety and effectiveness of vaccines plays an important role in an individual’s decision to get vaccinated [57]. The effectiveness and safety of mpox vaccine have been confirmed by several studies [9, 10]. Based on the results of our review and the study by León-Figueroa et al. [58], people who were confident in the safety and efficacy of the mpox vaccine were more likely to be vaccinated. Therefore, experts should develop and promote a global consensus on mpox vaccination. Mpox vaccine is offered primarily to people at high risk. For HIV-infected patients who are at risk for mpox infection, clinicians should make reasonable prophylactic and therapeutic recommendations based on the patient’s clinical status, viral suppression, and CD4 cell count [26].

The results showed that the pooled vaccine refusal rate among MSM was 16%. In addition to the above influencing factors, participants were more likely to refuse vaccines due to fear of privacy disclosure [18], stigma related to sexual orientation [59], and consideration of vaccination price [28, 60]. In the vaccine promotion work, healthcare workers and medical organizations should pay extra attention to privacy protection. Creating a more inclusive environment for sexual orientation can help eliminate resistance to vaccines among MSM. In addition, the free vaccination policy implemented by the government seems to be more conducive to MSM population acceptance of vaccination [61].

To the best of our knowledge, this is the first comprehensive meta-analysis of MSM willingness to receive mpox vaccine. To eliminate the risk of missing studies, we conducted a comprehensive literature search to determine the most recent estimates of vaccine intention rates. Sensitivity and subgroup analyses were also conducted to explore heterogeneous outcomes and identify potential sources of heterogeneity. As a result, the study design was enhanced. In addition, this study analyzed factors associated with vaccination willingness. This complements the shortcomings of other reviews on monkeypox vaccine willingness.

This review also has some limitations. The limitations are mainly related to the limitations of the included studies. First, all included studies applied cross-sectional survey. This limits us from making causal hypotheses. If possible, more rigorously designed cohort studies are needed to confirm our findings. Second, most of the included studies collected data by self-report, and there was no uniform assessment standard. This increases heterogeneity between studies. Experts should develop a common understanding of the measurement of vaccine willingness or hesitancy and develop an assessment tool that can be widely used. In addition, only papers written in English were included in this study. The generality of our findings should be carefully considered.

## Conclusion

MSM have a high willingness to vaccinate against mpox. Factors influencing vaccine willingness include behavioral characteristics, health status, knowledge of mpox and perceived associated risks, and confidence in the mpox vaccine. We recommended that public health authorities consider the identified factors when developing effective measures to promote vaccination among MSM. By understanding the unique needs and barriers faced by this population, public health officials can create targeted strategies to increase vaccine uptake. This proactive approach will better prepare authorities to prepare a scientific, evidence-based response plan for preventing and controlling future outbreaks of serious communicable diseases within the MSM population.

### Electronic supplementary material

Below is the link to the electronic supplementary material.

Appendix A

Appendix B

Appendix C

## Data Availability

Data available within the article or its supplementary materials.
