# Supplementary material for: Willingness to receive mpox vaccine among men who have sex with men: a systematic review and meta-analysis
Source: BMC Public Health. 2024 Jul 15;24:1878. doi: 10.1186/s12889-024-19260-9 (PMC11247826; doi:10.1186/s12889-024-19260-9)
Supplement: Supplementary file 1 — Appendix A [file 12889_2024_19260_MOESM1_ESM.docx]

**Pubmed: 107**

|  |  |
| --- | --- |
| #1 | "Sexual and Gender Minorities"[MeSH Terms] OR "Homosexuality"[MeSH Terms] OR "gay"[Title/Abstract] OR "men who have sex with men"[Title/Abstract] OR "MSM"[Title/Abstract] OR "LGBT"[Title/Abstract] OR "bisexual*"[Title/Abstract] OR "Queer"[Title/Abstract] OR "Gender Minority"[Title/Abstract] OR "Homosexual"[Title/Abstract] OR "non heterosexual*"[Title/Abstract] OR "Sexual Minority"[Title/Abstract] |
| #2 | "mpox (monkeypox)"[MeSH Terms] OR "Mpox virus"[MeSH Terms] OR "MPXV"[Title/Abstract] OR "human mpox"[Title/Abstract] |
| #3 | "vaccines"[MeSH Terms] OR "vaccination"[MeSH Terms] OR "vaccin*"[Title/Abstract] OR "immunis*"[Title/Abstract] OR "immuniz*"[Title/Abstract] |
| #4 | "accept*"[Title/Abstract] OR "attitude*"[Title/Abstract] OR "aware*"[Title/Abstract] OR "confiden*"[Title/Abstract] OR "willing*"[Title/Abstract] OR "uptake*"[Title/Abstract] OR "percept*"[Title/Abstract] OR "perceiv*"[Title/Abstract] OR "intent*"[Title/Abstract] OR "opinion*"[Title/Abstract] OR "knowledg*"[Title/Abstract] OR "ready"[Title/Abstract] OR "readiness"[Title/Abstract] OR "decid*"[Title/Abstract] OR "decision*"[Title/Abstract] OR "adher*"[Title/Abstract] OR "compliance"[Title/Abstract] OR "comply"[Title/Abstract] OR "trust*"[Title/Abstract] OR "choice*"[Title/Abstract] OR "choos*"[Title/Abstract] OR "belief*"[Title/Abstract] OR "literacy"[Title/Abstract] OR "practice"[Title/Abstract] OR "behavio*"[Title/Abstract] |
| #5 | "hesit*"[Title/Abstract] OR "delay*"[Title/Abstract] OR "doubt*"[Title/Abstract] OR "refus*"[Title/Abstract] OR "uncertain*"[Title/Abstract] OR "reluctan*"[Title/Abstract] OR "reservation*"[Title/Abstract] OR "reject*"[Title/Abstract] OR "sceptic*"[Title/Abstract] OR "distrust*"[Title/Abstract] OR "mistrust*"[Title/Abstract] OR "ambivalan*"[Title/Abstract] OR "unwilling*"[Title/Abstract] OR "concern*"[Title/Abstract] OR "oppos*"[Title/Abstract] OR "fear*"[Title/Abstract] OR "reject*"[Title/Abstract] OR "misinform*"[Title/Abstract] OR "object*"[Title/Abstract] OR "dilemma*"[Title/Abstract] OR "anxiet*"[Title/Abstract] OR "anxious*"[Title/Abstract] OR "worry"[Title/Abstract] OR "stigma"[Title/Abstract] OR "prejudic*"[Title/Abstract] OR "controvers*"[Title/Abstract] |
| #6 | #4 OR #5 |
| #7 | #1 AND #2 AND #3 AND #6 |

**Web of Science:176**

|  |  |
| --- | --- |
| #1 | TS=(“Sexual and Gender Minorities” or “gay” or “men who have sex with men” or “MSM” or “LGBT” or “Bisexual*” or “Queer” or “Gender Minority” or “Homosexual” or “Non-Heterosexual*” or “Sexual Minority”) and Preprint Citation Index (Exclude – Database) |
| #2 | **TS=("Mpox (monkeypox)" OR "Mpox virus" OR "MPXV" OR "human mpox")** and **Preprint Citation Index** (Exclude – Database) |
| #3 | TS=("vaccines" OR "vaccination" OR "vaccin*" OR "immunis*" OR "immuniz*") and Preprint Citation Index (Exclude – Database) |
| #4 | TS=(**“accept*" or "attitude*" or “aware*" or "confiden*" or "willing*" or "uptake*" or "percept*" or "perceiv*" or "intent*" or "opinion*" or "knowledg*" or "ready" or "readiness" or "decid*" or "decision*" or "adher*" or "compliance" or "comply" or "trust*" or "choice*" or "choos*" or "belief*" or "literacy" or "practice" or “behavio*”**) and Preprint Citation Index (Exclude – Database) |
| #5 | **TS=(****“hesit*” or “delay*” or “doubt*” or “refus*” or “uncertain*” or “reluctan*” or “reservation*” or “reject*” or “sceptic*” or “distrust*” or “mistrust*” or “ambivalan*” or “unwilling*” or “concern*” or “oppos*” or “fear*” or “reject*” or “misinform*” or “object*” or “dilemma*” or “anxiet*” or “anxious*” or “worry” or “stigma” or “prejudic*” or “controvers*”)** and **Preprint Citation Index** (Exclude – Database) |
| #6 | **#4 OR #5** and **Preprint Citation Index** (Exclude – Database) |
| #7 | **#1 AND #2 AND #3 AND #6** and **Preprint Citation Index** (Exclude – Database) |

**EMBASE:271**

|  |  |
| --- | --- |
| #1 | 'monkeypox'/exp |
| #2 | 'monkeypox virus'/exp |
| #3 | #1 OR #2 OR 'human mpox' OR 'mpox' OR 'mpxv' |
| #4 | 'vaccine'/exp |
| #5 | 'vaccination'/exp |
| #6 | #4 OR #5 OR 'vaccin*' OR 'immunis*' OR 'immuniz*' |
| #7 | 'men who have sex with men'/exp |
| #8 | 'bisexuality'/exp |
| #9 | 'sexual and gender minority'/exp |
| #10 | 'lgbtqia+ people'/exp |
| #11 | #7 OR #8 OR #9 OR #10 OR 'gay' OR 'msm' OR 'lgbt' OR 'bisexual*' OR 'queer' OR 'gender minority' OR 'homosexual' OR 'non-heterosexual*' OR 'sexual minority' |
| #12 | 'accept*':ti,ab,kw OR 'attitude*':ti,ab,kw OR 'aware*':ti,ab,kw OR 'confiden*':ti,ab,kw OR 'willing*':ti,ab,kw OR 'uptake*':ti,ab,kw OR 'percept*':ti,ab,kw OR 'perceiv*':ti,ab,kw OR 'intent*':ti,ab,kw OR 'opinion*':ti,ab,kw OR 'knowledg*':ti,ab,kw OR 'ready':ti,ab,kw OR 'readiness':ti,ab,kw OR 'decid*':ti,ab,kw OR 'decision*':ti,ab,kw OR 'adher*':ti,ab,kw OR 'compliance':ti,ab,kw OR 'comply':ti,ab,kw OR 'trust*':ti,ab,kw OR 'choice*':ti,ab,kw OR 'choos*':ti,ab,kw OR 'belief*':ti,ab,kw OR 'literacy':ti,ab,kw OR 'practice':ti,ab,kw OR 'behavio*':ti,ab,kw |
| #13 | 'hesit*':ti,ab,kw OR 'delay*':ti,ab,kw OR 'doubt*':ti,ab,kw OR 'refus*':ti,ab,kw OR 'uncertain*':ti,ab,kw OR 'reluctan*':ti,ab,kw OR 'reservation*':ti,ab,kw OR 'sceptic*':ti,ab,kw OR 'distrust*':ti,ab,kw OR 'mistrust*':ti,ab,kw OR 'ambivalan*':ti,ab,kw OR 'unwilling*':ti,ab,kw OR 'concern*':ti,ab,kw OR 'oppos*':ti,ab,kw OR 'fear*':ti,ab,kw OR 'reject*':ti,ab,kw OR 'misinform*':ti,ab,kw OR 'object*':ti,ab,kw OR 'dilemma*':ti,ab,kw OR 'anxiet*':ti,ab,kw OR 'anxious*':ti,ab,kw OR 'worry':ti,ab,kw OR 'stigma':ti,ab,kw OR 'prejudic*':ti,ab,kw OR 'controvers*':ti,ab,kw |
| #14 | #12 OR #13 |
| #15 | #3 AND #6 AND #11 AND #14 |

**CINAHL：73**

|  |  |
| --- | --- |
| S1 | TX ( "vaccination" or "vaccine" or "vaccin*" ) OR TX ( "immunis*" or "immuniz*" ) |
| S2 | TX ( “Mpox” or “monkeypox” or "monkey pox" ) OR TX ( "mpox virus" or MPXV" ) OR TX "human mpox" |
| S3 | TX (“Sexual and Gender Minorities” or “gay” or “men who have sex with men” or “MSM” or “LGBT” or “Bisexual*” or “Queer” or “Gender Minority” or “Homosexual” or “Non-Heterosexual*” or “Sexual Minority”) |
| S4 | TX (“accept*" or "attitude*" or “aware*" or "confiden*" or "willing*" or "uptake*" or "percept*" or "perceiv*" or "intent*" or "opinion*" or "knowledg*" or "ready" or "readiness" or "decid*" or "decision*" or "adher*" or "compliance" or "comply" or "trust*" or "choice*" or "choos*" or "belief*" or "literacy" or "practice" or “behavio*”) OR TX ( “hesit*” or “delay*” or “doubt*” or “refus*” or “uncertain*” or “reluctan*” or “reservation*” or “reject*” or “sceptic*” or “distrust*” or “mistrust*” or “ambivalan*” or “unwilling*” or “concern*” or “oppos*” or “fear*” or “reject*” or “misinform*” or “object*” or “dilemma*” or “anxiet*” or “anxious*” or “worry” or “stigma” or “prejudic*” or “controvers*”) |
| S5 | S1 AND S2 AND S3 AND S4 |

**Scopus:297**

|  |  |
| --- | --- |
| #1 | TITLE-ABS-KEY ( "sexual and gender minorities" OR "gay" OR "men who have sex with men" OR "msm" OR "lgbt" OR "bisexual*" OR "queer" OR "gender minority" OR "homosexual" OR "non-heterosexual*" OR "sexual minority" ) |
| #2 | TITLE-ABS-KEY ( "monkeypox" OR "mpox virus" OR "mpox" OR "mpxv" OR "human mpox" ) |
| #3 | TITLE-ABS-KEY ( "vaccines" OR "vaccination" OR "vaccin*" OR "immunis*" OR "immuniz*" ) |
| #4 | TITLE-ABS-KEY ( “accept*" or "attitude*" or “aware*" or "confiden*" or "willing*" or "uptake*" or "percept*" or "perceiv*" or "intent*" or "opinion*" or "knowledg*" or "ready" or "readiness" or "decid*" or "decision*" or "adher*" or "compliance" or "comply" or "trust*" or "choice*" or "choos*" or "belief*" or "literacy" or "practice" or “behavio*”) |
| #5 | TITLE-ABS-KEY ( **“hesit*” or “delay*” or “doubt*” or “refus*” or “uncertain*” or “reluctan*” or “reservation*” or “reject*” or “sceptic*” or “distrust*” or “mistrust*” or “ambivalan*” or “unwilling*” or “concern*” or “oppos*” or “fear*” or “reject*” or “misinform*” or “object*” or “dilemma*” or “anxiet*” or “anxious*” or “worry” or “stigma” or “prejudic*” or “controvers*”**) |
| #6 | (TITLE-ABS-KEY ( **“hesit*” or “delay*” or “doubt*” or “refus*” or “uncertain*” or “reluctan*” or “reservation*” or “reject*” or “sceptic*” or “distrust*” or “mistrust*” or “ambivalan*” or “unwilling*” or “concern*” or “oppos*” or “fear*” or “reject*” or “misinform*” or “object*” or “dilemma*” or “anxiet*” or “anxious*” or “worry” or “stigma” or “prejudic*” or “controvers*”** ) ) OR (TITLE-ABS-KEY ( accept* OR attitude* OR aware* OR confiden* OR willing* OR uptake* OR percept* OR perceiv* ORintent* OR opinion* OR knowledg* OR ready OR readiness OR decid* OR decision* OR adher* OR compliance OR complyOR trust* OR choice* OR choos* OR belief* OR literacy OR practice OR behavio* ) ) |
| #7 | #1 AND #2 AND #3 AND #6 |
