# Supplementary material for: Willingness to receive mpox vaccine among men who have sex with men: a systematic review and meta-analysis
Source: BMC Public Health. 2024 Jul 15;24:1878. doi: 10.1186/s12889-024-19260-9 (PMC11247826; doi:10.1186/s12889-024-19260-9)
Supplement: Supplementary file 2 — Appendix B [file 12889_2024_19260_MOESM2_ESM.docx]

**Results of the studies quality assessment**

| **Study** | **Items*** | | | | | | | | **Scores** | **Level of Evidence** | **Overall appraisal** |
| --- | --- | --- | --- | --- | --- | --- | --- | --- | --- | --- | --- |
|  | **1** | **2** | **3** | **4** | **5** | **6** | **7** | **8** |  |  |  |
| Reyes-Uruena et al. 2022 | N | Y | Y | Y | Y | Y | Y | Y | 7 | High | Include |
| Wang et al. 2022 | N | Y | Y | Y | N | N | Y | Y | 5 | Medium | Include |
| Zheng et al. 2022 | Y | Y | Y | Y | Y | Y | Y | Y | 8 | High | Include |
| Zucman et al. 2022 | N | Y | Y | N | Y | Y | N | Y | 5 | Medium | Include |
| Fu et al. 2023 | Y | Y | Y | Y | Y | Y | Y | Y | 8 | High | Include |
| Li et al. 2023 | Y | Y | Y | Y | Y | Y | Y | Y | 8 | High | Include |
| Dukers-Muijrers et al. 2023 | N | Y | Y | Y | Y | Y | Y | Y | 7 | High | Include |
| Hori et al. 2023 | N | Y | Y | N | Y | Y | N | Y | 5 | Medium | Include |
| Chow et al. 2023 | Y | Y | NC | NC | Y | Y | NC | Y | 5 | Medium | Include |
| MacGibbon et al. 2023 | Y | Y | Y | Y | NC | NC | Y | Y | 6 | Medium | Medium |
| Svartstein et al. 2023 | Y | Y | Y | Y | Y | Y | Y | Y | 8 | High | Include |
| Araoz-Salinas et al. 2023 | Y | Y | Y | Y | Y | Y | Y | Y | 8 | High | Include |
| Chen et al. 2023 | Y | Y | Y | Y | Y | Y | Y | Y | 8 | High | Include |
| Karapinar et al. 2023 | Y | N | Y | Y | Y | Y | Y | Y | 7 | High | Include |
| Zheng et al. 2023 | N | Y | Y | Y | N | N | Y | Y | 5 | Medium | Include |
| Smith et al. 2023a | Y | Y | Y | Y | Y | Y | Y | Y | 8 | High | Include |
| Jongen et al. 2023 | Y | Y | Y | Y | Y | Y | Y | Y | 8 | High | Include |
| Andersen et al. 2024 | Y | N | Y | Y | N | N | Y | Y | 5 | Medium | Include |
| Huang et al. 2024 | Y | Y | Y | Y | Y | Y | Y | Y | 8 | High | Include |
| Ogaz et al. 2024 | Y | Y | Y | Y | N | N | Y | Y | 6 | Medium | Include |
| *:Item 1: Were the criteria for inclusion in the sample clearly defined?  Item 2: Were the study subjects and the setting described in detail?  Item 3: Was the exposure measured in a valid and reliable way?  Item 4: Were objective, standard criteria used for measurement ofthe condition?  Item 5: Were confounding factors identified?  Item 6: Were strategies to deal with confounding factors stated?  Item 7: Were the outcomes measured in a valid and reliable way?  Item 8: Was appropriate statistical analysis used? | | | | | | | | | | |  |
|  |  |  |  |  |  |  |  |  |  |  |  |
|  |  |  |  |  |  |  |  |  |  |  |  |
|  |  |  |  |  |  |  |  |  |  |  |  |
|  |  |  |  |  |  |  |  |  |  |  |  |
|  |  |  |  |  |  |  |  |  |  |  |  |
|  |  |  |  |  |  |  |  |  |  |  |  |
|  |  |  |  |  |  |  |  |  |  |  |  |
|  |  |  |  |  |  |  |  |  |  |  |  |
