# Supplementary material for: Willingness to receive mpox vaccine among men who have sex with men: a systematic review and meta-analysis
Source: BMC Public Health. 2024 Jul 15;24:1878. doi: 10.1186/s12889-024-19260-9 (PMC11247826; doi:10.1186/s12889-024-19260-9)

1. **Heterogeneity test result。**

**
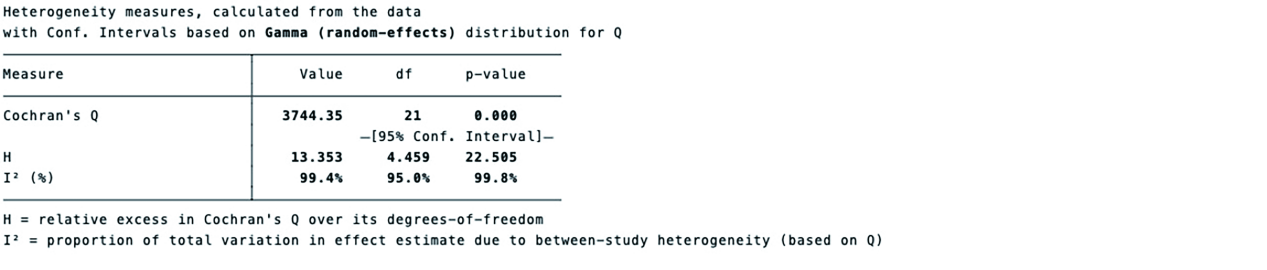
**

1. **The results subgroup analysis:**
   1. **Subgroup analysis of mpox vaccination willingness rates by countries.**

**
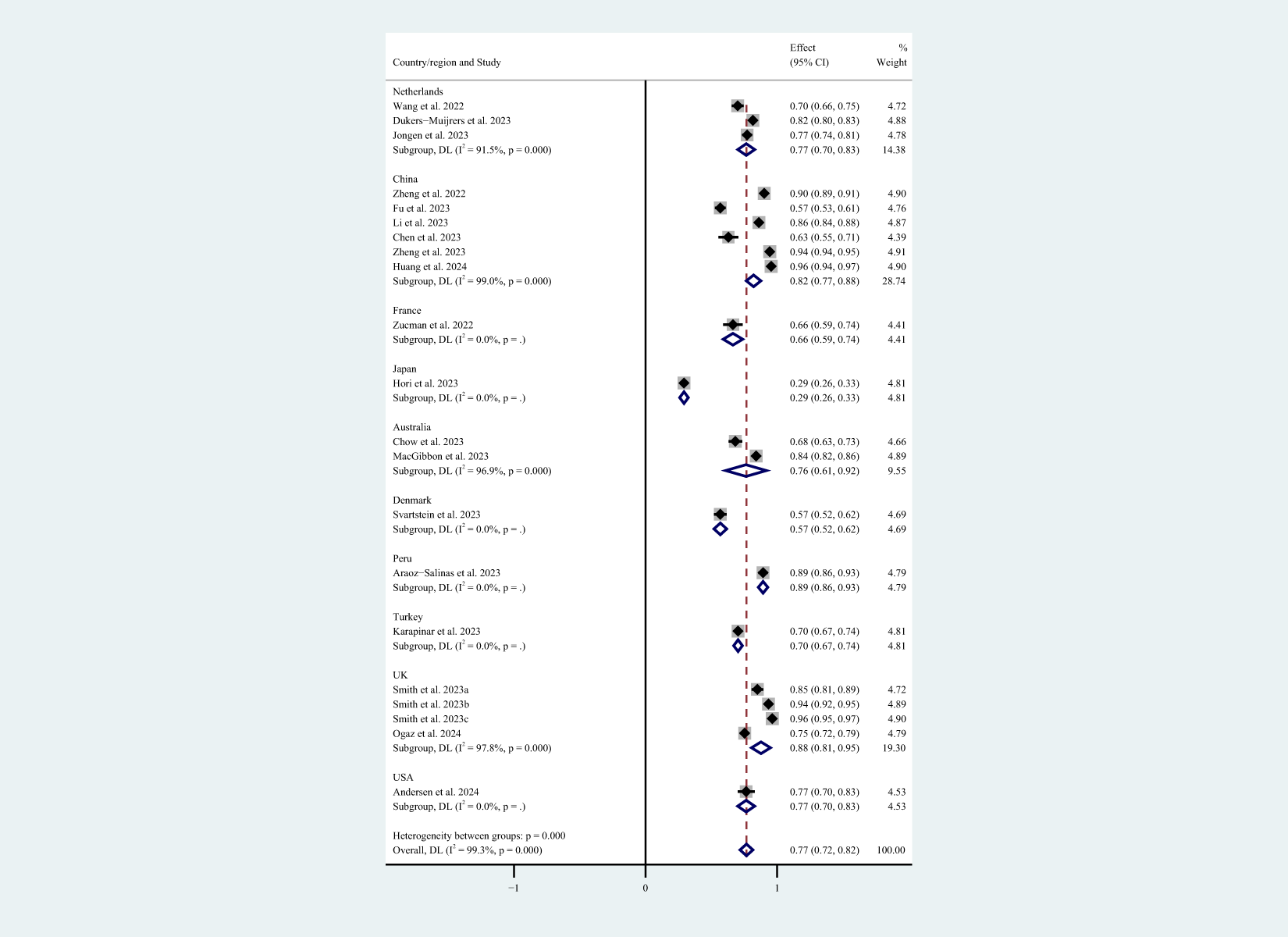
**

- 1. **Subgroup analysis of mpox vaccination willingness rates by sample size.**

**
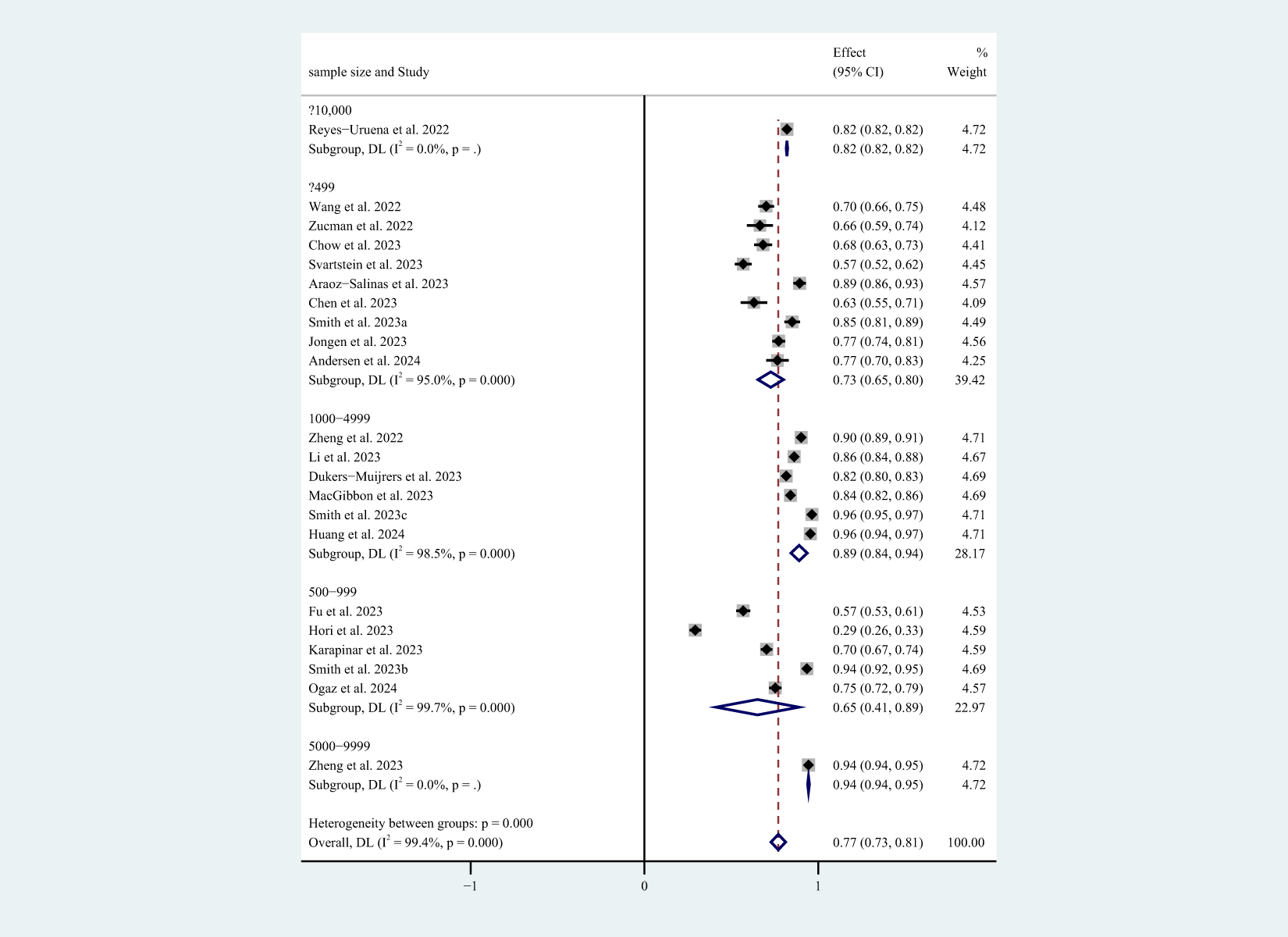
**

**2.3 Subgroup analysis of mpox vaccination willingness rates by survey date.**

**
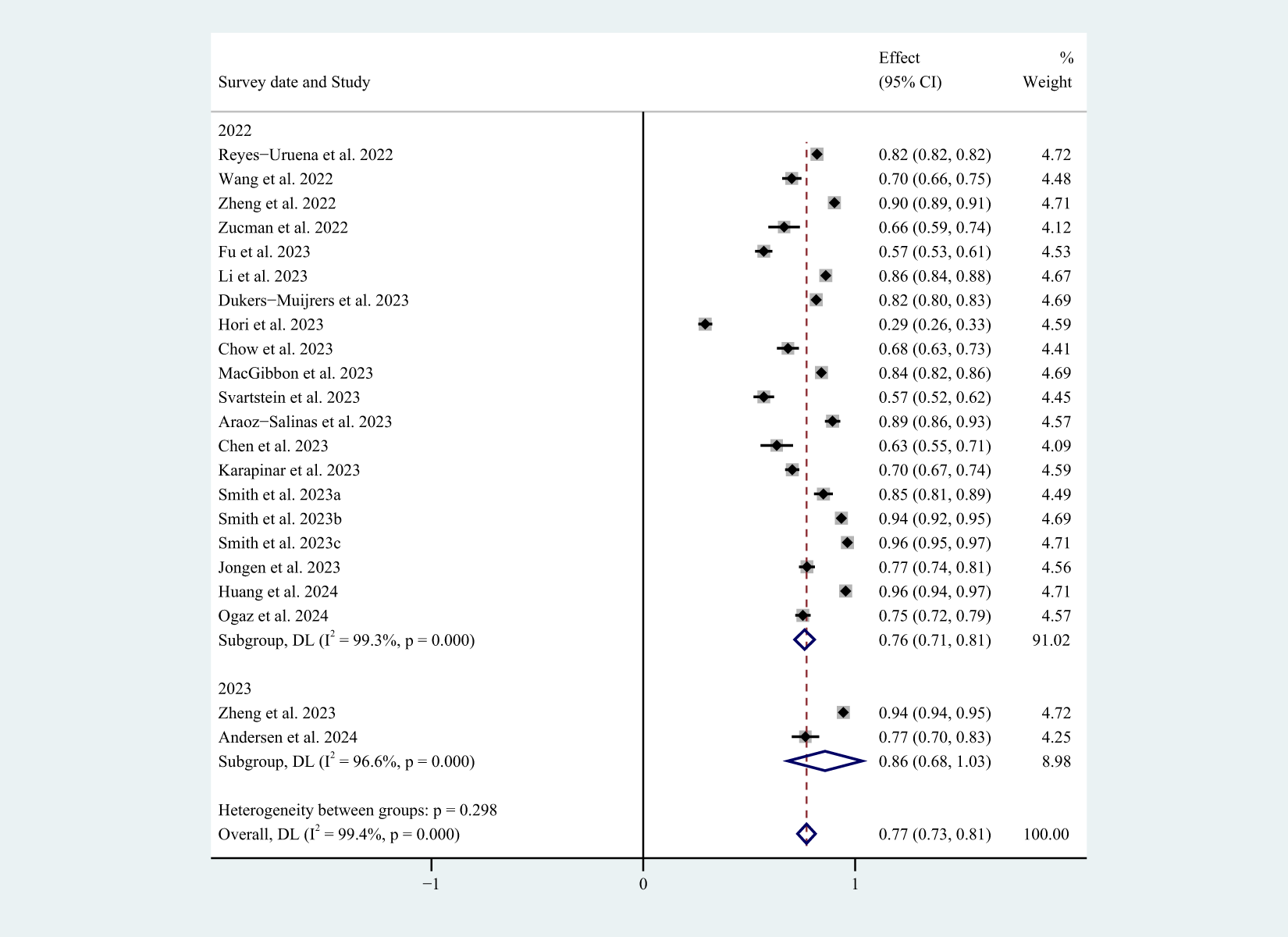
**

**2.4 Subgroup analysis of mpox vaccination willingness rates by continents.**

**
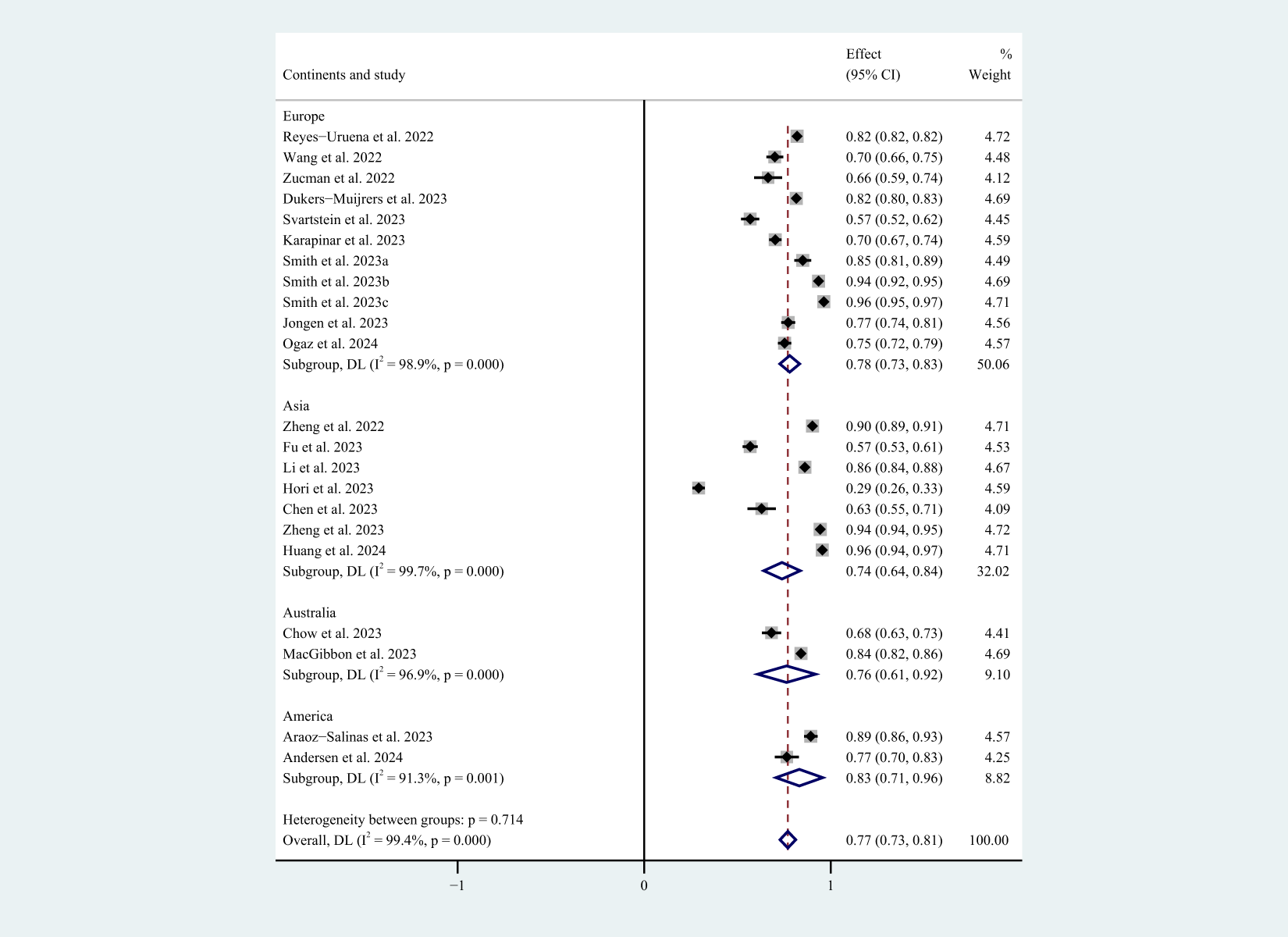
**

1. **Egger’s test.**

**
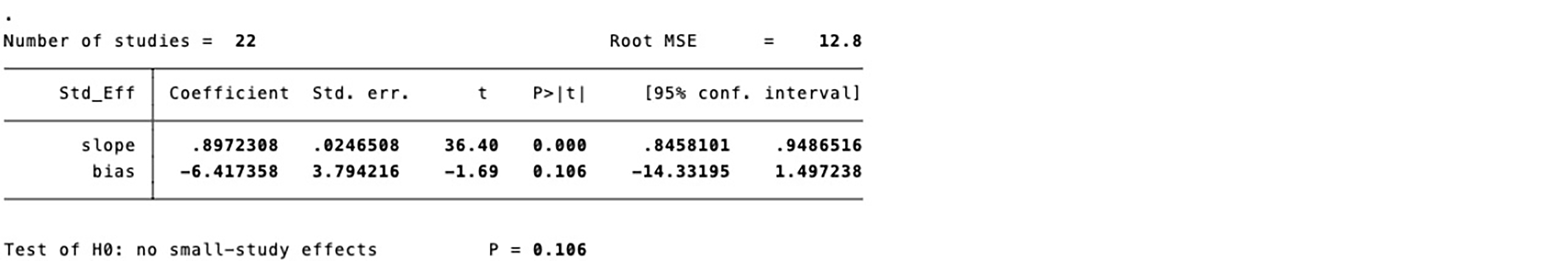
**

1. **Publication bias test.**

**
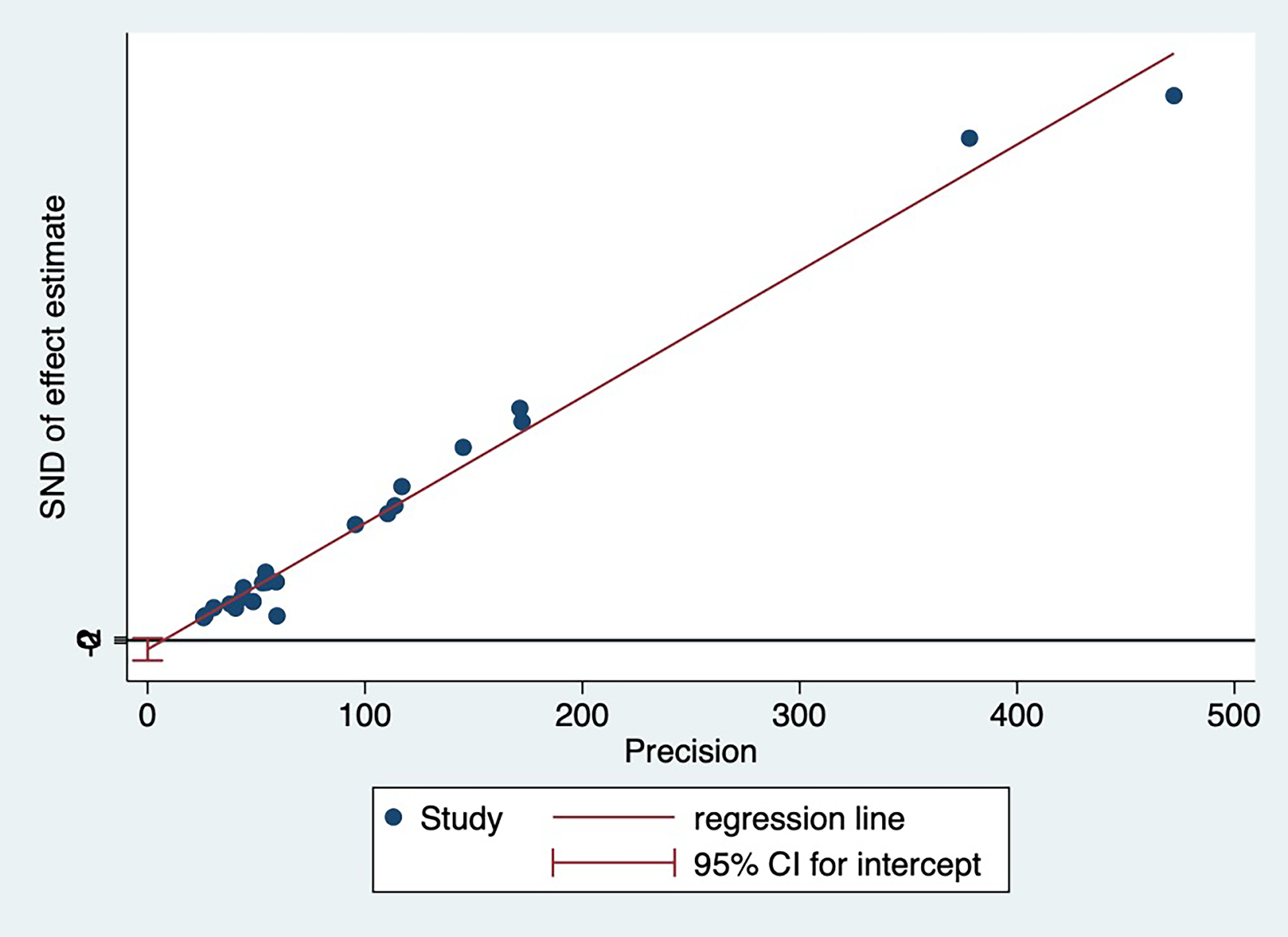
**

1. **Sensitivity analysis results.**


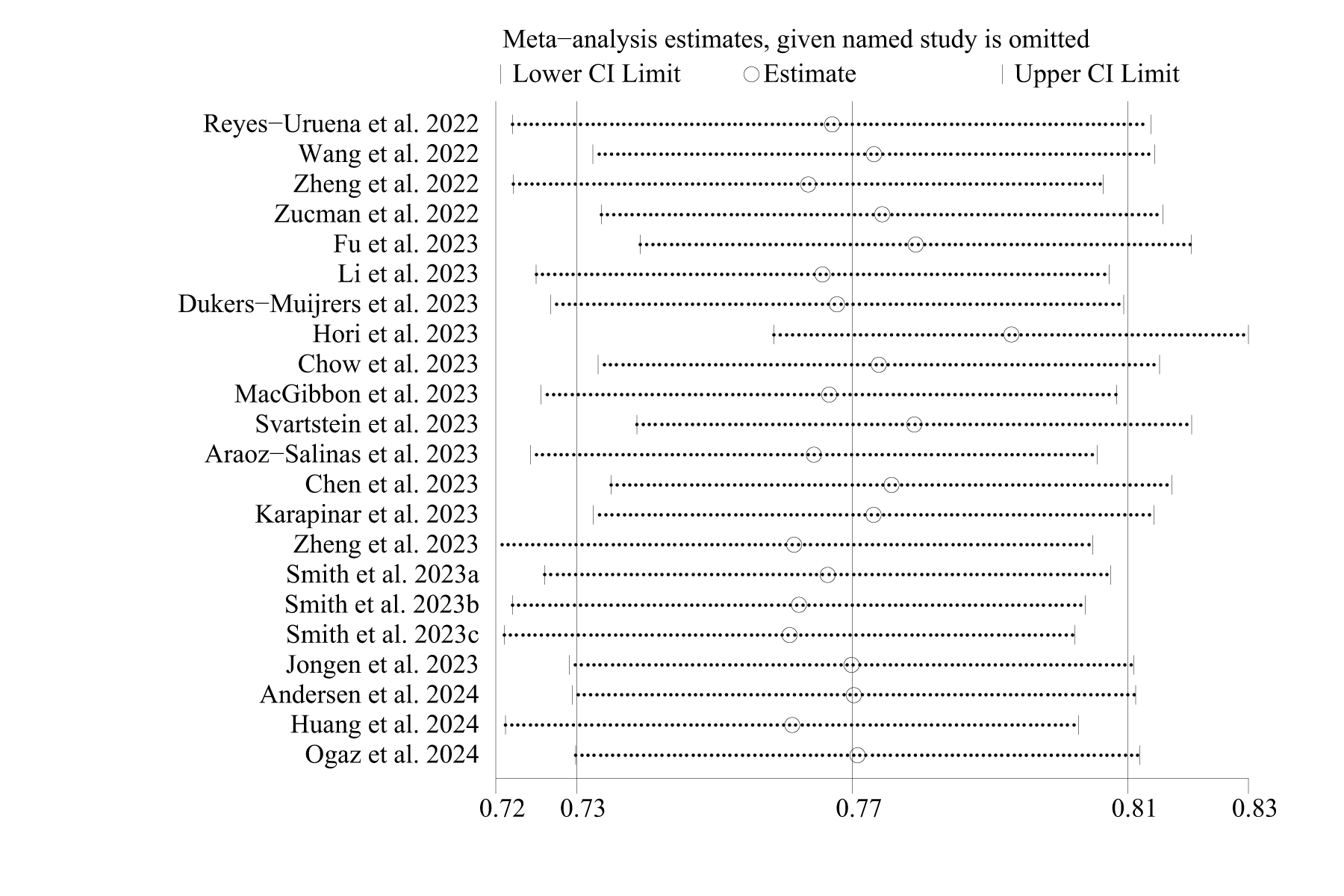

Supplement: Supplementary file 3 — Appendix C [file 12889_2024_19260_MOESM3_ESM.docx]
